# MOOCs and SMOCs: changing the face of medical education?

**DOI:** 10.1007/s40037-013-0103-y

**Published:** 2014-01-30

**Authors:** Neel Sharma, Iain Doherty, Darren Harbutt

**Affiliations:** Centre for Teaching and Learning, The University of Hong Kong, Hong Kong, China

Dear Sir,

The revolutionary forces at play in medical education are full steam ahead. From PBL, TBL and the flipped classroom, which has barely had time to cement itself we might add, we are now faced with the prospect of massive open online courses (MOOCs) and synchronous massive online courses (SMOCs).

MOOCs or massive open online courses are still fairly premature in the field of medicine with limited courses currently available. Examples in present use include Introductory Human Physiology by Coursera and Fundamentals of Clinical Trials courtesy of edX. From a learning perspective we can only hypothesize the potential benefits of such a movement. For instance, students may gain an enhanced understanding of pathology not common to their resident country or gain further understanding of hard to grasp concepts by clinicians renowned in their field of expertise.

Whilst medical schools are not currently offering academic credits for MOOC courses, the American Council on Education has certified four Coursera courses [1] so that students can gain credits recognized by institutes of higher education. At the same time Coursera has announced that MOOC courses can count for continuing medical education [1].

However, just like the flipped classroom, the dust has been barely left to settle following the arrival of MOOCs as a result of the recently announced SMOCs which may hold greater promise [2]. Gosling and Pennebaker, both psychology professors at the University of Texas in Austin, coined the term when they delivered their lectures live to enrolled students. Gosling’s interest is based on his stated downside of MOOCs being the lack of intimacy, community and simultaneous experience. From our perspective, such a concept could no doubt enhance medical learning in real time, adding to the concept of learning from live patient consultations and procedural intervention, which is currently a rare occurrence.

Whatever the future holds for medical education, it is sure to continue to amaze many an instructor and we for one feel it should be embraced wholeheartedly.
